# Cardamonin exerts anti-gastric cancer activity via inhibiting LncRNA-PVT1-STAT3 axis

**DOI:** 10.1042/BSR20190357

**Published:** 2019-05-17

**Authors:** Zheng Wang, Xiaoli Tang, Xiaoqing Wu, Meiyuan Yang, Wei Wang, Liuhua Wang, Dong Tang, Daorong Wang

**Affiliations:** 1 Department of General Surgery, Northern Jiangsu Province Hospital, Clinical Medical College, Institute of General Surgery – Yangzhou, Yangzhou University, Yangzhou, P.R. China; 2 Department of General Surgery, The Second Xiang ya Hospital of Central South University, Changsha, P.R. China

**Keywords:** Cardamonin, Gastric cancer, Inflammation, LncRNA-PVT1

## Abstract

**Background:** Gastric cancer is one of the most commonly diagnosed cancers each year, and it remains the third leading cause of cancer death in the world. The clinicopathologic characteristics differ among regions, so epigenetic changes play a key role in gastric carcinogenesis. **Methods:** In the present study, we first demonstrate that cardamonin, a natural production of chalcone, is an anti-gastric cancer agent in pre-clinical evaluation. **Results:** Cardamonin inhibited proliferation and migration, induced apoptosis in gastric cancer cells. It could reduce the expression of apoptosis-related and migration-related genes and proteins. The constant activation of STAT3 (signal transducer and activator of transcription 3) signal is a major intrinsic signal for cancer inflammation. It regulates cellular proliferation, cell cycle, and migration that are critical for cancer procession. Cardamonin could effectively down-regulate p-STAT3 and abolish activation of STAT3 through inhibiting the expression of LncRNA-PVT1. **Conclusion:** The present study revealed that cardamonin is a potential natural source of anti-gastric cancer drugs via epigenetic mechanism to inhibit LncRNA-PVT1-STAT3 axis.

## Introduction

Gastric cancer is a cancer which develops from the inner lining of the stomach. According to the World Health Organization (WHO), gastric cancer is the fifth most common cancer, but the third leading cause of cancer-related deaths each year all over the world [1]. The majority of people diagnosed with gastric cancer are a type referred to as adenocarcinoma of the stomach. The cancer develops from the cells that form the mucous membrane which is the most superficial lining of the stomach and produces mucus. Because the majority of people diagnosed with gastric cancer already at the advanced stage, patients often respond poorly to chemotherapy that causes long-term and serious side effects, resulting in a 5-year survival rate of less than 5%. Therefore, it is necessary to develop new and safe strategies with more potent anti-tumor efficiency as well as specificity against gastric cancer while reducing side effects.

Epigenetic mechanisms, which are traditionally defined as mitotically and/or meiotically heritable changes in gene expression without any modification of the primary DNA sequence, frequently occur in tumorgenesis and are potentially reversible. Epigenetic regulation, such as DNA methylation, histone modifications, noncoding RNAs expression, has been chosen as a promising molecular targets for cancer prevention. Environmental factors, including *Helicobacter pylori* and Epstein–Barr virus infection, high salt consumption, smoking, and erratic lifestyle, have an impact on gastric cancer incidence and contribute to the development of this disease [2,3]. Thus, the epigenetic regulation plays an important role in the gastric cancer formation and progression.

Long non-coding RNAs (LncRNAs) are defined as transcripts longer than 200 nucleotides without protein-coding capacity and exhibited poor sequence conservation. LncRNAs are involved in many biological and pathological functions during development, apoptosis, differentiation, aging, which regulate gene expression through miRNA sponge, the chromosomal dosage compensation, chromatin imprinting, chromatin modification, maintenance of chromatin structure, transcription, splicing, and translation [4,5]. Recent studies have demonstrated that LncRNA are differentially expressed in various human diseases, including cancer, cardiovascular disease, and Alzheimer’s disease [6–8]. So, LncRNA could be used in diagnosis, monitoring of progression, and targeted therapies in various diseases, and served as therapeutic targets for drug development [9].

The activating inflammatory signals can initiate or promote oncogenic transformation, and genetic and epigenetic changes in tumor cells [10]. Recent evidence suggests a crucial role of signal transducer and activator of transcription 3 (STAT3) is selectively induced and maintain a pro-carcinogenic inflammatory microenvironment during the cancer progression [11]. It is linked to inflammation-associated tumorigenesis that is initiated by chemical carcinogens, sunlight, infection, cigarette smoking, and stress [11].

**Figure 1:**
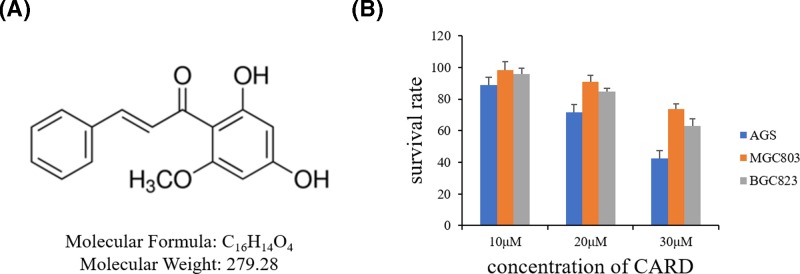
The chemical structure of CARD and its effect on apoptosis in human PC cell lines (**A**) The chemical structure of CARD and (**B**) the survival rates of the three human gastric cancer cell lines treated with increasing concentrations of CARD. Data represent the mean ± SD of three independent experiments.

Cardamonin (CARD, 2′,4′-dihydroxy-6′-methoxychalcone) (Figure 1A), which is a kind of chalcone, is a primary active ingredient and isolate from several plants including *Alpinia* [12]. It has been widely studied for its potential use in various types of disease, including pain, inflammatory bowel disease, cancer [13–15]. In this research, we found that CARD could inhibit human gastric cancer cell AGS and detect the underlying molecular mechanisms.

## Materials and methods

### Cells and culture

The human gastric cancer cell lines (AGS, MGC-803, BGC-823) were grown in RPMI-1640 supplemented with 10% fetal bovine serum, 100 U/ml penicillin, and 100 μg per streptomycin, and incubated at 37°C with 5% CO_2_.

### Reagents

CARD (purity ≥ 95% by HPLC) was purchased from Merck-Millipore Co, and was dissolved with DMSO and stored at –20°C. The antibody of Bax, Bcl-2, Caspase-3, E-cadherin, α-SMA, Vimentin, p21, CDC25c, Cyclin B1, CDK1 was purchased from Abcam, and Snail, p-STAT3, STAT3, and GAPDH from Cell Signaling Technology.

### Cell viability assay

Cell viability was measured by MTT assay. For MTT analysis, cells were incubated into 96-well plate and cultured for 24 or 48 h and then treated with or without different concentration of CARD. Cells were cultured with MTT in the incubator for 2 h. The formazan produced was dissolved in DMSO, and the absorbance at 540 nm was measured with a microplate reader.

### Colony formation assay

The AGS cells were seeded in 6-well plates at a density of 500 cells per well. After 2 weeks, plates were washed three times with PBS, fixed with 4% paraformaldehyde, and stained with Giemsa for 30 min at room temperature. Subsequent to washing with PBS, colonies with >50 cells were counted under an Lecia microscope.

### Sphere-forming assay

A total of 4 × 10^3^ cells were washed with PBS and resuspended in culture medium in ultra-low adherence 96-well plates (Corning). Spheres, >75 μM diameter, were counted after 7 days by light microscopy.

### Cell apoptosis analysis

AGS cultured in 12-well plates and treated with CARD. Then cells were collected and incubated at room temperature with an Annexin-V/propidium iodide (PI). The staining was performed according to the protocol. Finally, cell apoptosis was detected by flow cytometer.

### Cell cycle analysis

AGS cultured in 12-well plates and treated with CARD. Then cells were collected and fixed in ethanol at −20 °C overnight. Next day, the cells were washed by PBS and incubated with PI staining buffer at room temperature for 15 min and detected by flow cytometer.

### Dead/live assay

AGS cultured in 12-well plates and treated with CARD. Then cells were stained by LIVE/DEAD™ Cell Imaging Kit and followed the protocol.

### 2D scratch wound assay

The cells were cultured in the 6-wells plate, scratched by a 0.2 ml pipette tip, and the debris was removed. The cells were cultured with different concentrations of CARD for 24 h. Images were acquired by microscope and analyzed.

### Immunofluorescence analysis

AGS cells were cultured in 48-well plates and treated with CARD. Twenty-four hours later, the cell supernatants were cleared. Then the cells washed in PBS, and fixed in 4% paraformaldehyde for 20 min, and blocked for 30 min with 5% BSA and ruptured by 0.2% Trinton-100X, incubated with first and second antibodies. Then cells were performed using fluorescence microscope.

### RNA interference

For the treatment, siRNAs and scramble were purchased from GenePharma (GenePharma Co., Ltd., Shanghai, China). The sequences of selected regions to be targeted by siRNAs for PVT1 were taken from Takahashi et al. [16]. AGS cells were cultured in 6-well plates and then transfected with scramble, siRNA with Lipofectamine 3000 (Invitrogen, Carlsbad, CA, U.S.A.) according to the manufacturer’s instructions.

### Western blot analysis

AGS cells were collected, washed with cold PBS, and lysed with PhosphoSafe™ Extraction. We aspirated culture medium from cells, rinsed cells once with PBS. Then, the recommended amount of PhosphoSafe™ Extraction Reagent was added, incubated at room temperature for 5 min, scraped cell debris using a cell scraper, transferred the extract to 1.5 ml tube, and spined for 5 min at 16,000 × ***g*** at 4°C. At last, cell extract was transferred to a new tube and proceed with analysis. Proteins were electrophoresed using SDS-PAGE gel and transferred to the PVDF membranes. The membranes were blocked by 5% non-fat milk and were incubated with primary antibodies overnight at 4 °C. Then, the membranes were washed and incubated with the secondary antibodies at room temperature for 1 h. Finally, membranes were detected by imaging system.

### Statistical analysis

The data were expressed as mean  ±  standard deviation (SD). Student’s *t*-test was performed as appropriate using SPSS v20.0 software (IBM, Armonk, NY, U.S.A.). Significance was established at a *P* value of less than 0.05.

## Results

### Inhibition of gastric cancer cell proliferation by CARD

**Figure 2:**
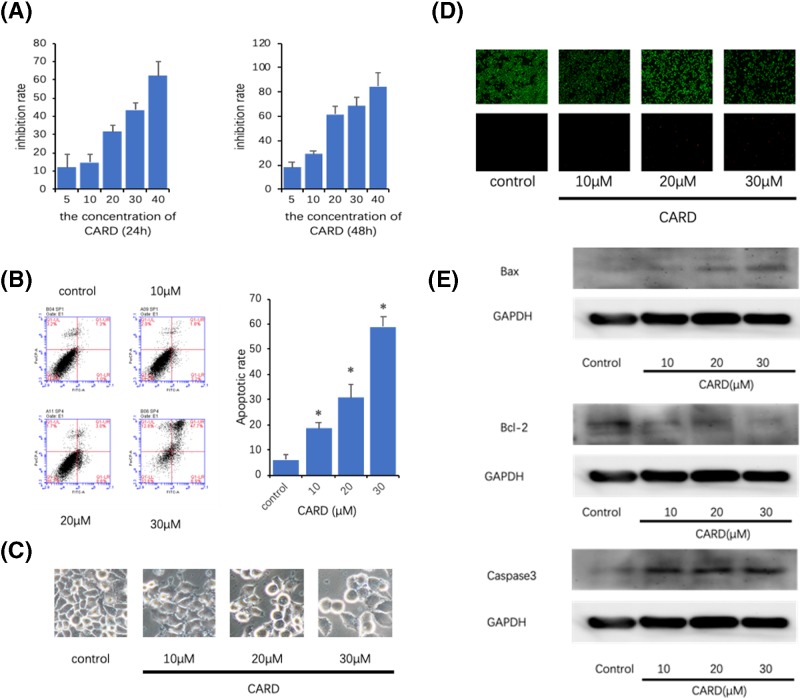
CARD kills gastric cancer cell lines (**A**) The inhibition rates of the human gastric cancer cell line treated with increasing concentrations of CARD in 24 or 48 h; (**B**) the apoptosis ratios were determined by Annexin V-FITC/PI staining; (**C**) microscopy was used to observe the cell morphology; (**D**) analysis of the dead and live cell by LIVE/DEAD™ Viability/Cytotoxicity Kit; (**E**) Western blot was used to measure Bcl-2, Bax, and Caspase-3 protein expression. Three such experiments were quantified by measuring the intensity of apoptosis-related proteins relative to the GAPDH (loading) control. Data represent the mean ± SD of three independent experiments. *P<0.05.

In the treatment of the gastric cancer cell lines, AGS was the most sensitive cancer cell line for CARD induced apoptosis (Figure 1B). So, AGS was used for the next research. AGS with CARD for 24 or 48 h exhibited inhibitory effect on the rate of proliferation. The cells were treated with different concentrations of CARD and then analyzed for proliferation (Figure 2A).

### Induction of apoptosis in gastric cancer cells by CARD

AGS cells were treated with different concentrations of AGS for 24 h and then stained using DEAD/LIVE kit or Annexin V-FITC and PI. An increase in the proportion of apoptotic cells was observed with the increase in concentration of CARD using fluorescence microscopy (Figure 2C,D). Flow cytometric assay was used to confirm the dose-dependent increase in the population of apoptotic cells by CARD (Figure 2B).

### Regulation of apoptosis relative protein expression by CARD

The western blot results showed that treatment with CARD down-regulated Bcl-2, and increased Bax protein expression (Figure 2E). Caspase-3 is a protein associated with cell apoptosis, and it is considered a tumor suppressor because it acts downstream of Bax/Bcl-2 control and plays a key role in the execution of apoptosis [17]. The result revealed that the protein expression levels of Caspase-3 in CARD group were significantly higher than the control group.

### Inhibition of gastric cancer cell colony formation by CARD

**Figure 3:**
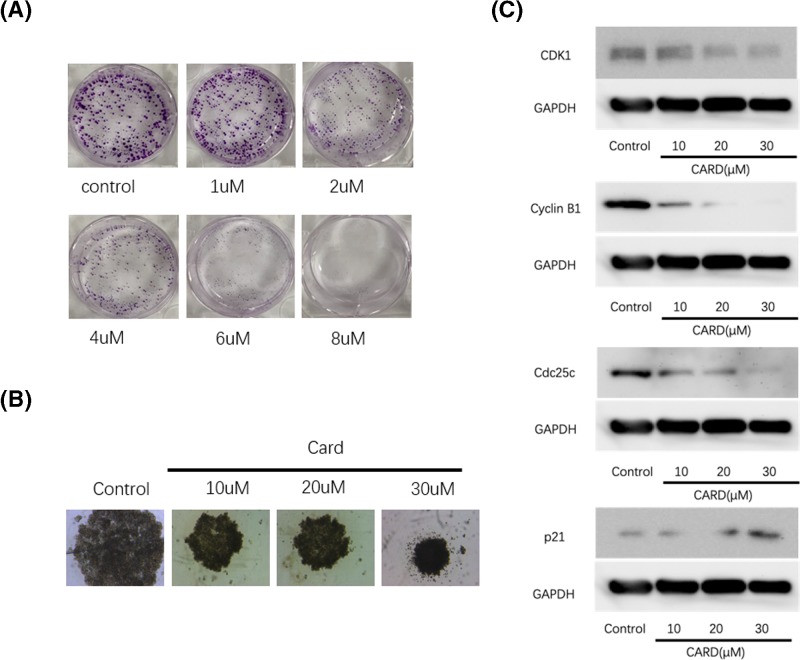
CARD inhibit gastric cancer cell lines (**A** and **B**) CARD inhibited cancer cell colony proliferation growth in 1 week; (**C**) Western blot was used to measure CDK1, Cyclin B1, CDC25c, and p21 protein expression. Four such experiments were quantified by measuring the intensity of cell cycle-related proteins relative to the GAPDH (loading) control.

The colony formation and sphere-forming assays are an *in vitro* technique to assay the neoplastic cells for clonogenic growth potential [18]. CARD induced a significant decrease in colony formation and sphere-forming of AGS cell lines (Figure 3A,B). The number and the size of colonies of the treatment group were less and smaller than the nontreatment groups.

### Regulation of proliferation relative protein expression by CARD

The western blot results showed that treatment with CARD down-regulated CDK1, Cyclin B1, and CDC25c while it increased p21 protein expression (Figure 3C). The result revealed that the CDK1, Cyclin B1 and CDC25 protein expression levels of in CARD treated group was significantly lower than the control group and the expression of p21 was opposite to the former three proteins. The interaction between CARD and these proteins indicated the potential proliferation inhibiting function of CARD in gastric cancer cell.

### Inhibition of gastric cancer cell migration by CARD

**Figure 4:**
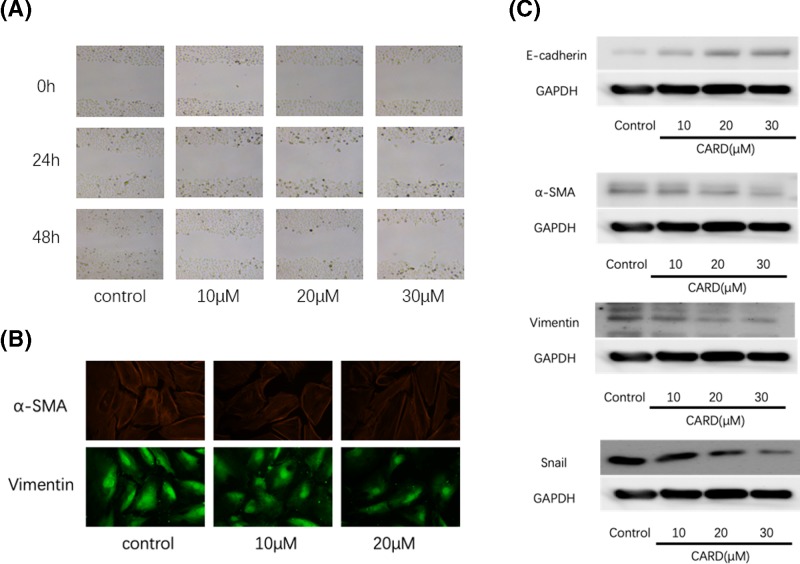
CARD inhibit gastric cancer cell lines immigration (**A**) CARD inhibited cancer cell immigration; (**B**) immunofluorescence was used to measure α-SΜΑ and Vimentin (**C**) Western blot was used to measure E-cadherin, α-SΜΑ, Vimentin, and Snail protein expression. Four such experiments were quantified by measuring the intensity of EMT-related proteins relative to the GAPDH (loading) control.

Migration is a major clinical characteristics for the high mortality and poor prognosis of gastric cancer [19]. As shown in Figure 4A, CARD suppresses the cancer cell migration by 2D scratch wound assay.

### Regulation of migration and invasion relative protein expression by CARD

Epithelial–mesenchymal transition (EMT) procession plays important roles in the tumor cell invasion and migration. As shown in Figure 4B,C, CARD enhanced the protein expression of E-cadherin (an epithelial marker), and decreased Snail, Vimentin, and α-SMA (a mesenchymal marker) protein expression. CARD suppresses the tumor invasion and metastasis in gastric cancer cell by regulating the EMT procession.

### Inhibition of STAT3 cell signal in AGS cell by CARD

**Figure 5:**
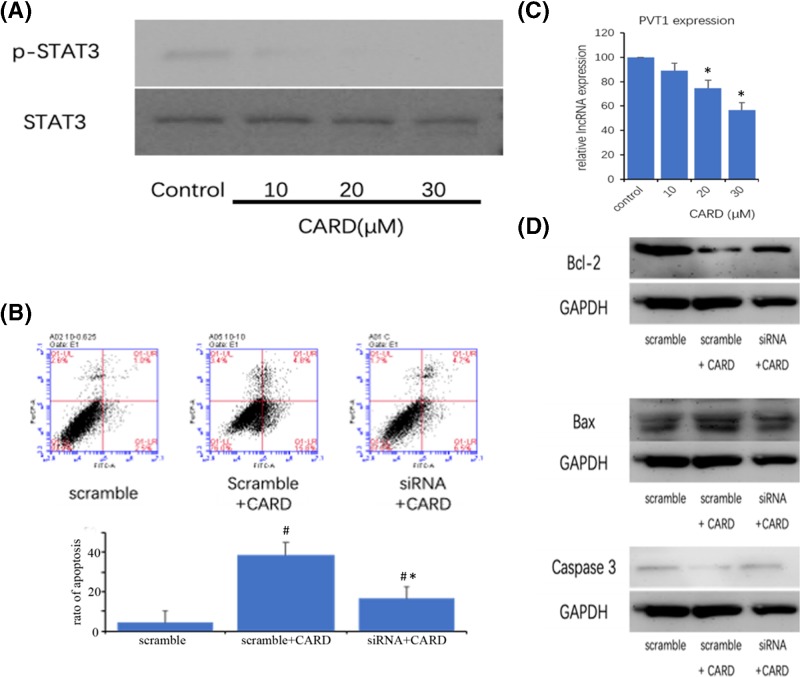
CARD inhibit gastric cancer cell lines STAT3 cell signal activate (**A**) CARD inhibited STAT3 phosphorylation; (**B**) CARD inhibit PVT1 LncRNA expression; (**C**) the apoptosis ratios were determined by Annexin V-FITC/PI staining; (**D**) Western blot was used to measure Bcl-2, Bax, and Caspase-3 protein expression. Three such experiments were quantified by measuring the intensity of apoptotic-related proteins relative to the GAPDH (loading) control. Data represent the mean ± SD of three independent experiments (^*,#^*P*<0.05).

STAT3, which is a central regulator of cancer cell proliferation and invasion, is activated by cytokines and growth factors via tyrosine phosphorylation (dimerization), and nuclear translocation in tumor cells [11,20]. Therefore, investigating the tyrosine phosphorylation of STAT3 was a useful method to discuss the STAT3 activation. As shown in Figure 5A, the phosphorylation level of STAT3 was suppressed by CARD in AGS cells.

### Inhibition of LncRNA-PVT1 in AGS cell by CARD

LncRNAs play crucial roles in cancer initiation, promotion, and progression [21]. PVT1 is one of the LncRNAs, which is highly expressed in some type of cancers and promotes cancer cell proliferation and stem cell-like property, and knockdown of PVT1 could weaken the resistance to doxorubicin and cisplatin [22–24]. Now, the study proved that PVT1 could directly interact with STAT3 in the nucleus, increase its protein stability, and activate the STAT3 signaling pathway. So, we detect the expression of PVT1. As shown in Figure 5B, CARD was associated with significantly reduced expression of PVT1. The cell apoptosis and apoptosis-related protein were significant inhibited by down-regulation of PVT1 (Figure 5C,D).

## Discussion

Persistent activation of STAT3 cell signal is a common characteristic of various cancers. CARD exhibits a variety of biological activities such as anti-inflammatory and anti-tumor, so we hypothesize that CARD exerts anti-tumor activity by regulating cancer-related LncRNA-PVT1-STAT3 axis.

Abnormal cell proliferation is one of the key features of malignant cells. So, arresting cell cycle progression and inducing cancer cell apoptosis are the potential mechanistic basis of most anti-cancer chemotherapeutic agents [25]. CARD blocked cell cycle progression at the G0/G1 phase and promoted apoptosis in the gastric cancer cell, indicating that it exerts its anti-tumor effects. Bcl-2 protein family determines the commitment of cells to trigger the mitochondrial suicide program apoptosis. This protein family includes the pro-apoptotic and anti-apoptotic proteins. Bcl-2 is considered as an anti-apoptotic protein that prevents cytochrome *C* release and inhibits the intrinsic apoptosis pathway. But Bax could form a pore in the mitochondrial membrane to allow cytochrome *C* release from mitochondria into the cytoplasm to Caspase-3 to trigger cell death. In the present study, CARD induced the AGS cell apoptosis, with a concomitant decrease the Bcl-2 protein expression and increase the Bax and Caspase-3 protein expression.

The depth of tumor invasion and lymph node metastasis are considered as the most important prognostic predictors of gastric cancer [26]. It is necessary to find the new treatment strategies and drugs that inhibit the invasion and metastasis for improving the survival rate for cancer patients. We used the 2D scratch wound healing assay to examine whether CARD affected AGS cells migration and invasion or not. With the increased concentration, CARD significantly reduces the rate of wound closure.

EMT, which is a complex process, plays a key role in cancer metastasis and invasion. Following CARD treatment, the protein expressions of various EMT markers, such as Vimentin, α-SMA, E-cadherin, and Snail were decreased or increased by western blotting and immunofluorescence. Together, these results suggest that CARD suppresses gastric cancer cell metastasis by regulating the EMT.

STAT3 is a pivotal regulator for various physiological functions, including cell proliferation, apoptosis, migration, and immune response. And STAT3 protein is central in determining whether immune response promotes or inhibits cancer in the tumor microenvironment. CARD has been reported to have anti-inflammatory via Toll-like receptors signaling pathways and inhibited NF-κB activation [27]. We have demonstrated that it has anti-tumor activity.

We therefore speculated that CARD may exert its anti-tumor activity by regulating the STAT3 signaling pathway. We found that CARD had inhibited the levels of phosphorylated STAT3 in AGS cells, which suggested that CARD prevents gastric cancer development through down-regulating the activation of STAT3 in AGS cells.

Recently, some studies proved that LncRNAs exert important roles in regulating various cancer behaviors via regulating intracellular signaling pathways. PVT1, which is associated with tumor progression and predicts recurrence, directly interacted with STAT3, increased its protein stability, and inhibited poly-ubiquitination and proteasome-dependent degradation. So, we detected whether CARD could decrease the expression of LncRNA-PVT1. Luckily, CARD could markedly inhibit LncRNA-PVT1 expression. Inhibition of LncRNA-PVT1 expression is a crucial mechanism of anti-cancer activity of CARD.

In summary, CARD is an active pharmacological ingredient isolated from *Alpinia katsumadai hayata*, a herb used in traditional Chinese medicine. Although the anti-inflammation effects of CARD have been observed, little is known about its effects in cancer, as well as the exact molecular mechanism. This is the first study to report that CARD suppressed proliferation of AGS cells by blocking cell proliferation and promoting apoptosis and inhibited the migration of AGS cell by reversing cancer cell EMT. Furthermore, we also provided experimental evidence of the mechanistic role of LncRNA-PVT1-STAT3 signaling pathway in mediating the effects of CARD, and our findings may provide a novel therapeutic strategy and future applications for gastric cancer therapy.

## Abbreviations

EMT: epithelial–mesenchymal transition
LncRNA: long non-coding RNA
MTT: 3-(4,5-dimethyl-2-thiazolyl)-2,5-diphenyl-2-H-tetrazolium bromide，Thiazolyl Blue Tetrazolium Bromide
PI: propidium iodide
PVDF: polyvinylidene fluoride
SD: standard deviation
STAT3: signal transducer and activator of transcription 3

## References

[1] Xu Z., Chen L., Xiao Z., Zhu Y., Jiang H., Jin Y. (2018) Potentiation of the anticancer effect of doxorubicinin drug-resistant gastric cancer cells by tanshinone IIA. Phytomedicine 51, 58–67 10.1016/j.phymed.2018.05.012 30466628

[2] Crew K.D. and Neugut A.I. (2006) Epidemiology of gastric cancer. World J. Gastroenterol. 12, 354–362 10.3748/wjg.v12.i3.354 16489633 PMC4066052

[3] Dicken B.J., Bigam D.L., Cass C., Mackey J.R., Joy A.A. and Hamilton S.M. (2005) Gastric adenocarcinoma: review and considerations for future directions. Ann. Surg. 241, 27–39 15621988 10.1097/01.sla.0000149300.28588.23 PMC1356843

[4] Mattick J.S. (2011) Long noncoding RNAs in cell and developmental biology. Semin. Cell Dev. Biol. 22, 327 10.1016/j.semcdb.2011.05.002 21621631

[5] Clark M.B. and Mattick J.S. (2011) Long noncoding RNAs in cell biology. Semin. Cell Dev. Biol. 22, 366–376 10.1016/j.semcdb.2011.01.001 21256239

[6] Zhou X. and Xu J. (2015) Identification of Alzheimer’s disease-associated long noncoding RNAs. Neurobiol. Aging 36, 2925–2931 10.1016/j.neurobiolaging.2015.07.015 26318290

[7] Haemmig S. and Feinberg M.W. (2017) Targeting LncRNAs in cardiovascular disease: options and expeditions. Circ. Res. 120, 620–623 10.1161/CIRCRESAHA.116.310152 28209793 PMC5325063

[8] Schmitt A.M. and Chang H.Y. (2016) Long noncoding RNAs in cancer pathways. Cancer Cell 29, 452–463 10.1016/j.ccell.2016.03.010 27070700 PMC4831138

[9] Bhat S.A., Ahmad S.M., Mumtaz P.T., Malik A.A., Dar M.A., Urwat U. (2016) Long non-coding RNAs: mechanism of action and functional utility. Noncoding RNA Res. 1, 43–50 10.1016/j.ncrna.2016.11.002 30159410 PMC6096411

[10] Mantovani A., Allavena P., Sica A. and Balkwill F. (2008) Cancer-related inflammation. Nature 454, 436–444 10.1038/nature07205 18650914

[11] Yu H., Pardoll D. and Jove R. (2009) STATs in cancer inflammation and immunity: a leading role for STAT3. Nat. Rev. Cancer 9, 798–809 10.1038/nrc2734 19851315 PMC4856025

[12] Lee J.H., Jung H.S., Giang P.M., Jin X., Lee S., Son P.T. (2006) Blockade of nuclear factor-kappaB signaling pathway and anti-inflammatory activity of cardamomin, a chalcone analog from Alpinia conchigera. J. Pharmacol. Exp. Ther. 316, 271–278 10.1124/jpet.105.092486 16183703

[13] Sambasevam Y., Omar Farouk A.A., Tengku Mohamad T.A., Sulaiman M.R., Bharatham B.H. and Perimal E.K. (2017) Cardamonin attenuates hyperalgesia and allodynia in a mouse model of chronic constriction injury-induced neuropathic pain: possible involvement of the opioid system. Eur. J. Pharmacol. 796, 32–38 10.1016/j.ejphar.2016.12.020 27988285

[14] Li Y., Qin Y., Yang C., Zhang H., Li Y., Wu B. (2017) Cardamonin induces ROS-mediated G2/M phase arrest and apoptosis through inhibition of NF-kappaB pathway in nasopharyngeal carcinoma. Cell Death Dis. 8, e3024 10.1038/cddis.2017.407 29048425 PMC5596588

[15] Wang K., Lv Q., Miao Y.M., Qiao S.M., Dai Y. and Wei Z.F. (2018) Cardamonin, a natural flavone, alleviates inflammatory bowel disease by the inhibition of NLRP3 inflammasome activation via an AhR/Nrf2/NQO1 pathway. Biochem. Pharmacol. 155, 494–509 10.1016/j.bcp.2018.07.039 30071202

[16] Takahashi Y., Sawada G., Kurashige J., Uchi R., Matsumura T., Ueo H. (2014) Amplification of PVT-1 is involved in poor prognosis via apoptosis inhibition in colorectal cancers. Br. J. Cancer 110, 164–171 10.1038/bjc.2013.698 24196785 PMC3887297

[17] Yang B., Johnson T.S., Thomas G.L., Watson P.F., Wagner B., Furness P.N. (2002) A shift in the Bax/Bcl-2 balance may activate caspase-3 and modulate apoptosis in experimental glomerulonephritis. Kidney Int. 62, 1301–1313 10.1111/j.1523-1755.2002.kid587.x 12234300

[18] Wang Y.J., Bailey J.M., Rovira M. and Leach S.D. (2013) Sphere-forming assays for assessment of benign and malignant pancreatic stem cells. Methods Mol. Biol. 980, 281–290 10.1007/978-1-62703-287-2_15 23359160

[19] Lei C., Du F., Sun L., Li T., Li T., Min Y. (2017) miR-143 and miR-145 inhibit gastric cancer cell migration and metastasis by suppressing MYO6. Cell Death Dis. 8, e3101 10.1038/cddis.2017.493 29022908 PMC5682659

[20] Liu L., McBride K.M. and Reich N.C. (2005) STAT3 nuclear import is independent of tyrosine phosphorylation and mediated by importin-alpha3. Proc. Natl. Acad. Sci. U.S.A. 102, 8150–8155 10.1073/pnas.0501643102 15919823 PMC1149424

[21] Fang Y. and Fullwood M.J. (2016) Roles, functions, and mechanisms of long non-coding RNAs in cancer. Genomics Proteomics Bioinformatics 14, 42–54 10.1016/j.gpb.2015.09.006 26883671 PMC4792843

[22] Wan L., Sun M., Liu G.J., Wei C.C., Zhang E.B., Kong R. (2016) Long noncoding RNA PVT1 promotes non-small cell lung cancer cell proliferation through epigenetically regulating LATS2 expression. Mol. Cancer Ther. 15, 1082–1094 10.1158/1535-7163.MCT-15-0707 26908628

[23] Wang F., Yuan J.H., Wang S.B., Yang F., Yuan S.X., Ye C. (2014) Oncofetal long noncoding RNA PVT1 promotes proliferation and stem cell-like property of hepatocellular carcinoma cells by stabilizing NOP2. Hepatology 60, 1278–1290 10.1002/hep.27239 25043274

[24] Kun-Peng Z., Xiao-Long M. and Chun-Lin Z. (2018) Overexpressed circPVT1, a potential new circular RNA biomarker, contributes to doxorubicin and cisplatin resistance of osteosarcoma cells by regulating ABCB1. Int. J. Biol. Sci. 14, 321–330 10.7150/ijbs.24360 29559849 PMC5859477

[25] Grutter M.G. (2000) Caspases: key players in programmed cell death. Curr. Opin. Struct. Biol. 10, 649–655 10.1016/S0959-440X(00)00146-9 11114501

[26] Deng J.Y. and Liang H. (2014) Clinical significance of lymph node metastasis in gastric cancer. World J. Gastroenterol. 20, 3967–3975 10.3748/wjg.v20.i14.3967 24744586 PMC3983452

[27] Kim Ah-Yeon, Shim Hyun-Jin and Kim. Su Yeon (2018) Differential regulation of MyD 88- and TRIF-dependent signaling pathways of Toll-like receptors by cardamonin. Int. Immunopharmacol. 64, 1–9 10.1016/j.intimp.2018.08.018 30142469

